# Objective clinical registration of tremor, bradykinesia, and rigidity during awake stereotactic neurosurgery: a scoping review

**DOI:** 10.1007/s10143-024-02312-4

**Published:** 2024-02-14

**Authors:** Annemarie Smid, Zeus T. Dominguez-Vega, Teus van Laar, D. L. Marinus Oterdoom, Anthony R. Absalom, Martje E. van Egmond, Gea Drost, J. Marc C. van Dijk

**Affiliations:** 1 Department of Neurosurgery, University Medical Center Groningen, University of Groningen, Hanzeplein 1 HPC AB71, 9713 GZ Groningen, Netherlands; 2 Department of Neurology, University Medical Center Groningen, University of Groningen, Hanzeplein 1 HPC AB71, 9713 GZ Groningen, Netherlands; 3 Department of Anesthesiology, University Medical Center Groningen, University of Groningen, Hanzeplein 1 HPC AB71, 9713 GZ Groningen, Netherlands

**Keywords:** Motor symptoms, Intraoperative monitoring, Accelerometry, IMU, EMG, Optical measurements

## Abstract

Tremor, bradykinesia, and rigidity are incapacitating motor symptoms that can be suppressed with stereotactic neurosurgical treatment like deep brain stimulation (DBS) and ablative surgery (e.g., thalamotomy, pallidotomy). Traditionally, clinicians rely on clinical rating scales for intraoperative evaluation of these motor symptoms during awake stereotactic neurosurgery. However, these clinical scales have a relatively high inter-rater variability and rely on experienced raters. Therefore, objective registration (e.g., using movement sensors) is a reasonable extension for intraoperative assessment of tremor, bradykinesia, and rigidity. The main goal of this scoping review is to provide an overview of electronic motor measurements during awake stereotactic neurosurgery. The protocol was based on the PRISMA extension for scoping reviews. After a systematic database search (PubMed, Embase, and Web of Science), articles were screened for relevance. Hundred-and-three articles were subject to detailed screening. Key clinical and technical information was extracted. The inclusion criteria encompassed use of electronic motor measurements during stereotactic neurosurgery performed under local anesthesia. Twenty-three articles were included. These studies had various objectives, including correlating sensor-based outcome measures to clinical scores, identifying optimal DBS electrode positions, and translating clinical assessments to objective assessments. The studies were highly heterogeneous in device choice, sensor location, measurement protocol, design, outcome measures, and data analysis. This review shows that intraoperative quantification of motor symptoms is still limited by variable signal analysis techniques and lacking standardized measurement protocols. However, electronic motor measurements can complement visual evaluations and provide objective confirmation of correct placement of the DBS electrode and/or lesioning. On the long term, this might benefit patient outcomes and provide reliable outcome measures in scientific research.

## Introduction

Tremor, bradykinesia, and rigidity are debilitating motor symptoms that can be alleviated with stereotactic neurosurgical treatment [1, 2]. Currently, intraoperative neurosurgical decisions are based, in part, on subjective and episodic evaluations of tremor, bradykinesia, and rigidity [3–13]. Objective registration is a reasonable extension for intraoperative assessment of these cardinal symptoms [3, 4, 6, 7, 9, 11, 14–20]. Unfortunately, motor symptoms remain difficult to measure objectively, due to the heterogeneity of the symptoms between patients. Currently, expert evaluation is often used as the gold standard in research aiming to validate objective methods. Studies performed outside the intraoperative setting showed that electronic measurements can improve clinical evaluation, resulting in for example a decrease in scoring error of up to 20% [21], increased reliability [13, 22–24], and increased accuracy [9, 22, 25–27]. These results motivate further investigation of objective intraoperative registration of these motor symptoms. However, studies on intraoperative motor measurements are scarce and rarely focused on tracing the course of motor symptoms during surgery [11, 19, 28–30]. Also, there are inconsistencies in the methods and protocols that are described. As a result, objective and quantitative input is still lacking in the mainstream clinical decision-making for guiding and adjusting treatment. This paper aims to present a structured overview of current objective clinical registration of tremor, bradykinesia, and rigidity during awake stereotactic neurosurgery, to discuss the key findings in the current literature, and to identify trends that can be relevant for clinical practice.

### Clinical background

Tremor, bradykinesia, and rigidity can occur in several movement disorders, like Parkinson’s disease (PD), essential tremor (ET), dystonic tremor (DT), and Holmes tremor (HT). While the sole motor symptom in ET is tremor, patients with PD also suffer from bradykinesia and rigidity [31, 32]. Tremor can also occur in dystonia, which is a movement disorder characterized by sustained or intermittent muscle contractions [33, 34]. HT is a low frequency, more proximal tremor syndrome, frequently caused by an acquired lesion in the brainstem [31].

Open neurosurgical treatment options for motor symptom reduction consist of deep brain stimulation (DBS) and radiofrequency (RF) needle ablation (e.g., thalamotomy (thal) and pallidotomy (pal)) [1]. Based on specific symptoms, potential targets include the subthalamic nucleus (STN), ventral intermediate nucleus (VIM), and globus pallidus internus (GPi) [2, 33, 35, 36], among others. Incisionless approaches include application of gamma knife [37] or magnetic resonance imaging (MRI)–guided focused ultrasound (MRgFUS) [28, 38].

During procedures under local anesthesia, immediate alleviation of motor symptoms can usually be observed after placement of the DBS electrode, RF-lesioning probe, or during the MRgFUS test lesion: the microlesion or stunning effect [2, 34, 39–41]. Several studies describe how the intensity of the stunning effect can be an indication of correct targeting [34, 39–41]. The magnitude of the intraoperative effect on the symptoms can be real-time monitored during awake procedures [38, 42, 43]. Sammartino et al. described the importance of intraoperative monitoring of motor symptoms and side effects for confirming optimal lead placement and predicting postoperative motor improvement [29].

Tremor, bradykinesia, and rigidity are often intraoperatively evaluated with disease-specific clinical rating scales, e.g., the Movement Disorder Society Unified PD Rating Scale (MDS-UPDRS) [44] and the Fahn–Tolosa–Marin Clinical Rating Scale for Tremor (FTM) [45]. As such, severity of symptoms is subjectively assessed by an expert [3–11, 16, 20], and its results depend on the rater’s experience, which may result in significant bias [3–11, 16]. For example, the results from Post et al. showed a rather large intra-rater variation and considerable inter-rater disagreement [8]. They also showed that if patients are evaluated by two different raters, the total UPDRS ratings can differ up to 16 points [8]. Different caregivers with different expertise levels are involved with rating the patient during the preoperative, intraoperative, and postoperative measurements, resulting in inter-rater variability. This lack in continuity in measurements might negatively affect patient outcomes [3–11, 16].

### Technical background

Wearable devices and video-based measurements allow for collection of data representing movement information of tremor, bradykinesia, and rigidity [46]. The use of such devices has been proven to aid experts in objectively quantifying symptoms during application of clinical protocols (e.g., MDS-UPDRS) [11, 14, 15, 17, 19]. This might reduce inter- and intra-rater variability and optimize clinical outcome. Nonetheless, devices have to fulfill various requirements in order to be suitable for human movement analysis, for example, small size, light weight, unobtrusive, reliable, and accurate [47]. Electromyography (EMG), accelerometers, gyroscopes, and optical devices that satisfy these requirements have already been validated for this purpose.

#### Tremor

The diagnosis of tremor is generally confirmed using EMG by measuring electrical activity of muscles [31, 48–50]. Electrical signals of the measured muscle groups are amplified and filtered to remove noise and interference. Depending on the application, information can be analyzed using time-domain techniques for amplitude and duration analysis, or using frequency-domain techniques involving the frequency content of the signals [51]. This technique has good reliability when compared to clinical scales such as the MDS-UPDRS [52].

Accelerometry is also used to assess tremor, by measuring linear movement-induced accelerations relative to the gravitational field [47, 53, 54]. The acceleration signal is transformed using frequency analysis to discriminate tremor from other activities. Voluntary movement is generally concentrated at frequencies below 2 Hz, while most tremor subtypes have a dominant frequency of 3 Hz or greater [31, 50]. Accelerometric data can be used to derive meaningful information about tremor, like frequency, amplitude, and constancy [11, 30, 46, 47, 53–55].

Gyroscopes are similarly suitable for tremor registration, especially for rotational PD tremor (“pill-rolling” tremor) [56]. Gyroscopes detect angular velocity of a rotating body by measuring the Coriolis force generated in a rotating reference frame [57, 58]. These sensors measure the rate of rotation of an object around one, two, or three axes [58]. Similar to previous techniques, gyroscopic data is transformed to be analyzed in the frequency domain to obtain amplitude and dominant frequency as metrics to evaluate tremor presence, intensity, and frequency [59–61].

#### Bradykinesia

The most commonly used tasks to assess hand bradykinesia include finger tapping, opening/closing of the fist, and pronation/supination of the arm, as described in the MDS-UPDRS [44]. Quantification of slowness, hesitancy, and movement amplitude decrements can be measured with EMG [49, 51, 62], accelerometers [49, 63], gyroscopes [56], and optical measurements [64–66]. Data from these devices is analyzed using various techniques like frequency analysis and velocity estimation, to quantify the severity of bradykinesia [23, 56, 62–67]. Strong associations between electronic measures and the clinical gold standard references (e.g., MDS-UPDRS) were reported for bradykinesia, indicating that the reliability of these techniques suffices clinical requirements [68]. Quantification of bradykinesia commonly employs a combination of sensors, in contrast to tremor being reliably quantified with a single sensor. A combination of accelerometers, gyroscopes, and magnetic sensors, known as inertial measurement unit (IMU), is the most commonly used technology, reporting a moderate correlation or an acceptable receiver operating characteristic (ROC) compared to gold standard references [68].

#### Rigidity

Rigidity is generally evaluated with clinical tests (e.g., MDS-UPDRS) where slow passive flexion and extension of the joint of interest is performed by the examiner to feel the resistance of the passive movement (“cogwheel phenomenon”) [44, 49]. Rigidity can usually be increased when a voluntary activation maneuver (e.g., fist opening/closing, or heel tapping) is performed by the contralateral limb [44, 49]. The amount and pattern of muscle activity during the movement can be evaluated based on EMG analysis [22, 49, 69, 70]. The outcome measures obtained using this technique include amount of resistance, uniformity of resistance, muscle activity amplitude, co-activation, pattern of muscle activity, and symmetry of muscle activity [22, 49, 69, 70]. These metrics have shown to have good reliability (intraclass correlation coefficient (ICC) 0.9) compared to clinical evaluation [22].

IMUs are also used to assess rigidity, by capturing, e.g., the range of movement, angular velocity, linear acceleration, and orientation of body segments during movement [22, 49, 71, 72]. Possible IMU outcome measures are joint angle, angular velocity, linear acceleration, jerk, and resistance to movement [22, 49, 71, 72]. Good reliability (ICC 0.8) can be found compared to clinical evaluation [22].

## Methods

### Protocol

The protocol was based on PRISMA Extension for Scoping Reviews (PRISMA-ScR) [73].

### Eligibility criteria

Peer-reviewed journal articles published in English were included. Eligible studies included electronic motor measurements in patients suffering from tremor, bradykinesia, and/or rigidity during awake stereotactic neurosurgery. Only human studies with an experimental or quasi-experimental design were included. Exclusion criteria were animal studies, (systematic) review articles, and conference abstracts. As this review focuses on electronic measurement of symptomatology, indirect measurement techniques, such as microelectrode recordings (MER), local field potentials (LFP), imaging, ultrasound, and intracerebral microvascular measurements, were not taken into account.

### Search strategy

Key terms from relevant articles were extracted to build the search string, which was tested and refined accordingly. PubMed (Medline, PubMed Central), Embase, and Web of Science were searched. No limit was applied for the publication date. The final search in all databases was performed on December 6, 2023. The result of each search was exported to Mendeley. As an example, the search string from PubMed is added to Appendix 1.

### Source selection

Each title and abstract were screened by the first and second author for relevance. Any disagreements were resolved. Papers that passed the first screening underwent a full-text review and were categorized as either included or excluded.

## Results

**Fig. 1:**
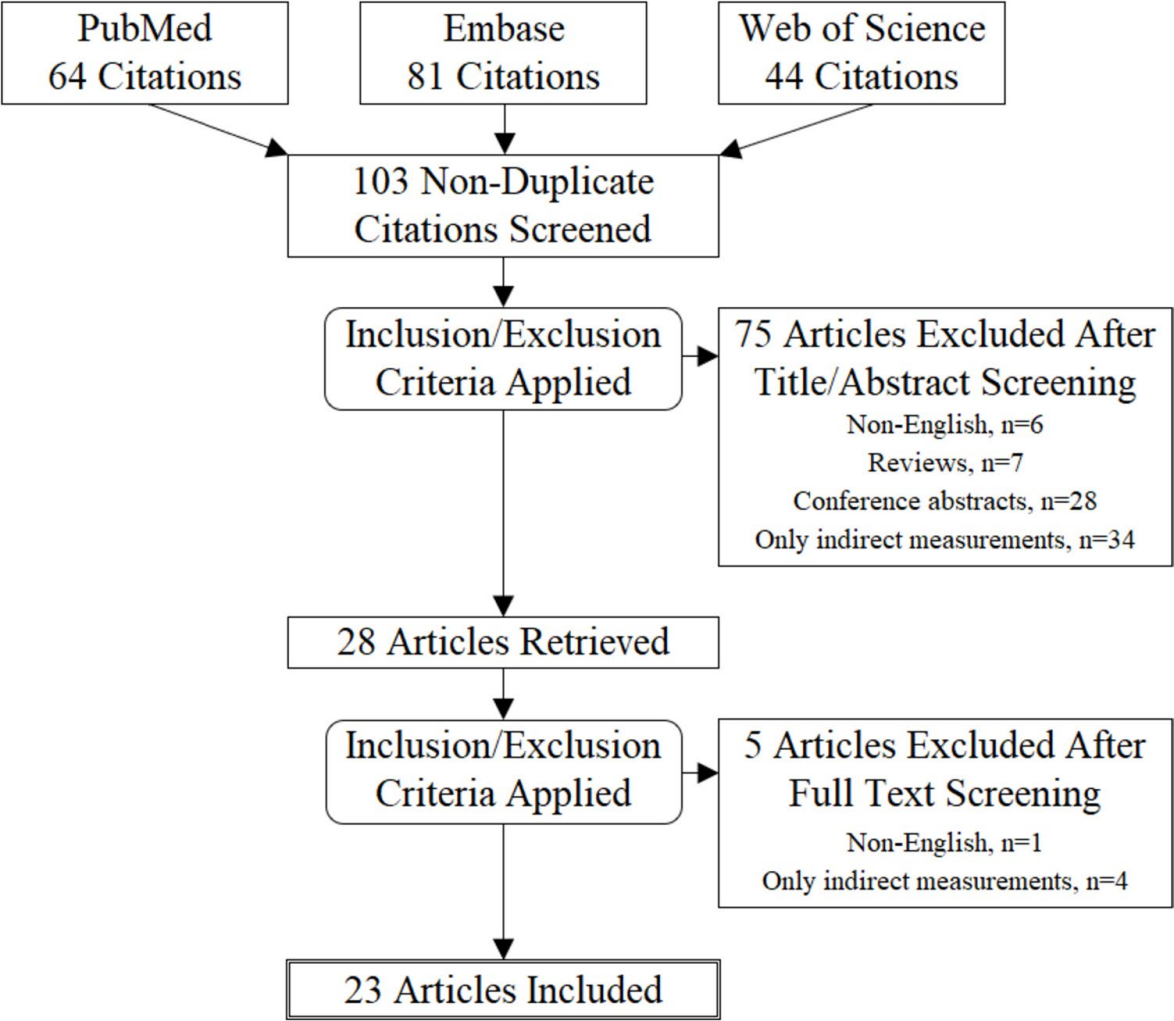
Search results and selection of articles

After screening and full-text review, 23 articles were included in the final review (Fig. 1). The number of subjects and controls, clinical diagnoses, symptoms, and surgery specifications of the included studies are shown in Table 1. The results are summarized in Tables 2, 3, and 4 [74–96].

**Table 1:** Overview of included studies

Authors, year [reference]	Participants					Symptoms			Surgery			Laterality		Targets		
	PD	ET	DT	HT	HC	Tremor	Bradykinesia	Rigidity	DBS	Thal	Pal	Uni	Bi	STN	VIM	GPi
Landy et al., 2000 [74]	21					21		21			21	21				21
Koop et al., 2006 [75]	61				15		61		61			24	37	61		
Papapetropoulos et al., 2008 [76]	12				10	12	12		12			12		12		
Papapetropoulos et al., 2009 [77]		1				1			1			1			1	
Waldau et al., 2011 [78]	8				5		8		8			4	4	5	3	
Florin et al., 2012 [79]	14					8			14				14	14		
Kwon et al., 2014 [80]	8							8	8			2	6	8		
Hemm et al., 2016 [81]		5				5			5				5		5	
Florin et al., 2016 [82]	26					8			26				26	26		
Shah et al., 2017 [83]	15					15			15				15	7	8	
Shah et al., 2017 [84]	9							9	9				9	9		
Milosevic et al., 2018 [85]	2	9				11			11			1	10		11	
Schaeffer et al., 2018 [86]	26	11				37		26	37			37			37	
Shah et al., 2020 [87]		5				5			5			1	4		5	
Wang et al., 2020 [88]	39					39			39			39		35		4
Múrias Lopes et al., 2020 [89]	59							59	59				59	59		
Wu et al., 2021 [90]	7						7		7				7	7		
Kremer et al., 2021 [91]			1			1			1				1		1	
Smid et al., 2022 [92]	28				26	28			22	6		6	22	22	6	
Yu et al., 2022 [93]	20				3		20		20				20	20		
Baek et al., 2023 [94]	9	9				18				18		18			18	
Smid et al., 2023 [95]	13	7		2		22				24		24			24	
Glowinsky et al., 2023 [96]		1				1			1				1		1

**Table 2:** Overview of the intraoperative studies on objectively measuring tremor

Device type	Location of the device	Outcome measure	References
Accelerometer	Held in the hand	Tremor amplitude (cm)	Papapetropoulos et al., 2008 (*n* = 12) [76], Papapetropoulos et al., 2009 (*n* = 1) [77]
		Center frequency (Hz)	
		SD of center frequency (frequency dispersion)	
		Harmonic index (HI)	
	Wrist	Standard deviation	Hemm et al., 2016 (*n* = 5) [81], Shah et al., 2017 (*n* = 15) [83], Shah et al., 2020 (*n* = 5) [87]
		Signal energy	
		Amplitude of dominant frequency	
		Scalar sum of accelerations	Milosevic et al., 2018 (*n* = 11) [85]
		Tremor reduction (%)	
	Hand dorsum	Acceleration (g)	Schaeffer et al., 2018 (*n* = 37) [86]
		Average signal power (g^2^)	
		Tremor frequency (Hz)	
	Index finger	Tremor frequency (Hz)	Smid et al., 2022 (*n* = 28) [92], Smid et al., 2023 (*n* = 22) [95]
		Tremor amplitude (cm)	
		Area-under-the-curve of power in 4–6 Hz range	
		Percentage of time that tremor was present (%)	Smid et al., 2022 (*n* = 28) [92]
	Arm, forearm, and index finger	Joint angle reduction (%)	Baek et al., 2023 (*n* = 9) [94]
Gyroscope	Hand dorsum	Angular velocity (dps)	Schaeffer et al., 2018 (*n* = 37) [86]
		Average signal power (dps^2^)	
sEMG	Several muscle groups	EMG response	Landy et al., 2000 (*n* = 21) [74]
	m. extensor digitorum communis m. flexor digitorum superficialis	Frequency (Hz)	Florin et al., 2012 (*n* = 14) [79], Florin et al., 2016 (*n* = 26) [82]
		Coherence (with LFP)	
	e. digitorum f. digitorum superficialis m. tibialis anterior m. gastrocnemius	Weighted Root Mean Square per second	Wang et al., 2020 (*n* = 39) [88]
		Modified Mean Absolute Value	
		Waveform Length	
		Zero Crossings	
		Slope Sign Changes	
		Sample Entropy	
		Peak Frequency (Hz)	
		Peak Frequency Power	
		Median Amplitude Power	
		Mean Amplitude Power	
		Frequency Ratio	
	m. sternocleidomastoideus m. semispinalis cervicis	EMG-burst activity (μV)	Kremer et al., 2021 (*n* = 1) [91]
		EMG-burst length (ms)	
	wrist flexor and extensor	EMG-burst activity (μV)	Glowinsky et al., 2023 (*n* = 1) [96]
		Frequency (Hz)	
		Neuronal/EMG coherence	
		Flexor–extensor cross-correlation

**Table 3:** Overview of the intraoperative studies on objectively measuring bradykinesia

Device type	Location of the device	Outcome measure	References
Gyroscope	Hand dorsum	Root mean square of angular velocity (deg/sec)	Koop et al., 2006 (*n* = 61) [75]
		Frequency (Hz)	
Optical device	In front of the patient	Velocity (units/s)	Waldau et al., 2011 (*n* = 8) [78]
		Reaction time (ms)	
	Around the patient	Distance (mm) between thumb and index finger	Wu et al., 2021 (*n* = 7) [90], Yu et al., 2022 (*n* = 20) [93]
		Distance (mm) finger tips and hand palm	
		Roll angle (°) of the hand palm	
		Frequency (Hz)	
		Velocity (mm/s)	
Touch sensor	In front of the patient	Maximal frequency (Hz)	Papapetropoulos et al., 2008 (*n* = 12) [76]

**Table 4:** Overview of the intraoperative studies on objectively measuring rigidity

Device type	Location of the device	Outcome measure	References
Accelerometer	Hand dorsum	Acceleration (g)	Schaeffer et al., 2018 (*n* = 37) [86]
		Average signal power (g^2^)	
	Wrist of the examiner	Standard deviation	Shah et al., 2017 (*n* = 9) [84]
		Signal energy	
		Amplitude of dominant frequency	
		Inertia calculation	Kwon et al., 2014 (*n* = 8) [80]
Torque sensor	Wrist of the examiner	Resistive torque integrated by angle (W) per cycle	
		Resistive torque integrated by time (I) per cycle	
		Mechanical impedance (Z)	
Potentiometer	Wrist of the examiner	Wrist angle (°)	
Gyroscope	Hand dorsum	Angular velocity (dps)	Schaeffer et al., 2018 (*n* = 37) [86]
		Average signal power (dps^2^)	
	Hand palm	Angular velocity of the wrist (°/s)	Múrias Lopes et al., 2020 (*n* = 59) [89]
sEMG	Several muscle groups	EMG response	Landy et al., 2000 (*n* = 21) [74]

### Demographics

Mean age and standard deviation of the included patients were only described in 14 of the included studies [74–77, 79, 80, 82, 88, 90–93, 95, 96] and the disease duration in 10 studies [75–77, 79, 80, 82, 90, 91, 95, 96]. For the studies that did mention these demographics, age ranged from 48 to 83 years, and the disease duration ranged from 3 to 35 years. Only one DT patient [91] and two HT patients [95] were described. Few studies included healthy participants (Table 1) [75, 76, 78, 92, 93].

### Main research aims

The principal aim of most studies was to objectively quantify change in tremor, bradykinesia, or rigidity during stereotactic neurosurgery [74–77, 80, 83, 84, 86, 87, 89, 90, 92–95]. Few studies specifically aimed to validate their approaches on electronic measurement of tremor, bradykinesia, or rigidity intraoperatively [80, 86, 93]. Kremer et al. aimed to show how EMG can be used to quantify dystonic tremor reduction during VIM-DBS surgery [91]. The aim of Hemm et al. [81] and Shah et al. [87] was to optimize DBS electrode positioning. Other studies were focused to quantify stimulation effects [78, 85, 88, 96]. Florin et al. aimed to intraoperatively identify causality patterns of tremor in two types of PD patients, by using surface EMG (sEMG) [79, 82].

### Clinical protocols

Most studies used tasks from the motor section of the MDS-UPDRS as their clinical intraoperative protocol [75, 76, 86, 88–90, 92, 93, 95]. Others did not use a standardized clinical scale intraoperatively [74, 78, 79, 81, 82, 85, 91, 96]. Papapetropoulos et al. used FTM as a clinical protocol to intraoperatively assess tremor [77]. Baek et al. used postural tremor tasks, signature writing, Archimedes spiral, and point-to-point drawing to assess tremor intraoperatively [94]. Four studies used a modified MDS-UPDRS to rate tremor and rigidity [80, 83, 84, 87]. Eleven out of 23 studies compared the electronic measurement data with a clinical scale, showing correlations ranging from − 0.76 to 0.99 (*p* < 0.001) [13, 75, 80, 83, 86, 88–90, 92–94]. Several studies focused on creating an objective method for measuring clinical outcome measures with visually scored scales [75, 76, 86, 90, 92, 93]. Smid et al. [92] aimed to translate a clinical scale to an objective scoring method, using internationally standardized MDS-UPDRS thresholds.

### Technical aspects

#### Tremor

Of 16 included tremor-studies, 10 used accelerometry [76, 77, 81, 83, 85–87, 92, 94, 95], and 6 used sEMG [74, 79, 82, 88, 91, 96]. Only Shaeffer et al. [86] used both accelerometer and gyroscope data.

Frequency analysis was the most common technique to assess tremor, since 13 [74, 76, 77, 79, 81–83, 85–88, 92, 95] out of 16 included studies used this to quantify tremor characteristics, mostly with the classical fast Fourier transform (FFT) [76, 77, 81, 83, 86–88]. Baek et al. did not mention their analysis technique [94]. Kremer et al. and Glowinsky et al. used raw sEMG data to investigate EMG burst activity by observation [91, 96]. The periodogram [92, 95] and coherence analysis [79, 82] have also shown good performance.

Apart from frequency domain analysis, time domain analysis of the acceleration or angular velocity was also used to quantify tremor characteristics. Papapetropoulos et al. [76, 77] and Smid et al. [92, 95] used the acceleration signal to estimate the position and calculated the amplitude of tremor in centimeters. Hemm et al. [81] and Shah et al. [83, 87] used the magnitude of the acceleration signal to estimate tremor intensity.

In all 16 included studies, different clinical tremor characteristics were considered outcome measures of the electronic assessment. Eight studies objectively quantified tremor frequency [76, 77, 81, 83, 86–88, 95], nine studies quantified amplitude [76, 77, 81, 83, 85, 87, 88, 92, 95], three quantified duration [85, 88, 92], two quantified intensity [76, 77], and eight quantified irregularity [76, 77, 79, 82, 88, 91, 96]. All studies took the presence of tremor into account [74, 76, 77, 79, 81–83, 85–88, 92, 95].

Most studies concluded that their quantitative approach might improve intraoperative monitoring or awake stereotactic targeting [74–78, 80, 81, 83–95]. It differed per study why this was the conclusion. None of the included studies presented sufficient data to conclude that electronic measurements would improve monitoring or stereotactic neurosurgery. Only Shah et al. investigated whether the intraoperative neurosurgical decision would be different based on their measurements: 15 of the 26 decisions would have been different. They were not able to judge whether the expert-based or the accelerometry-based electrode position would have been the best clinical outcome [83, 84]. Still, they did show the potential of triaxial accelerometry complementing tremor assessment during neurosurgery [83, 84]. Use of accelerometry to quantify change in tremor was reported to be superior with regard to traditional visual evaluation of tremor [76, 77, 81, 83, 85–87, 92, 94, 95].

The added value of EMG for intraoperative tremor quantification was demonstrated in all six tremor EMG studies [74, 79, 82, 88, 91, 96]. Wang et al. concluded that amplitude, motor unit firing rate, and changes in the regularity pattern could be used as quick intraoperative biomarkers to quantify and predict efficacy of DBS in PD-patients with resting tremor [88]. Kremer et al. concluded that the easy-to-use objective technique of polymyography can facilitate optimization of DBS-electrode placement [91].

#### Bradykinesia

Of the five included studies that measured bradykinesia, three studies used optical devices [78, 90, 93]. Papapetropoulos et al. [76] used a touch recording plate and Koop et al. [75] used a tri-axial gyroscope. Wu et al. [90] and Yu et al. [93] used a leap motion controller (LMC) to assess bradykinetic movements, extracting information of the position and orientation of the hands during different tasks. Waldau et al. used an optical virtual reality (VR) glove to estimate the velocity of hand movements [78]. Common outcome measures were velocity estimation [75, 78, 90, 93], reaction time [78], movement duration [78], position [90], orientation [90], rhythm [76], movement amplitude [90], and movement variability [93].

Koop et al. demonstrated a promising gyroscope-based method to quantify changes in upper extremity bradykinesia during different stages of DBS surgery [75]. Papapetropoulos et al. showed that maximal frequency FT as measured by the touch recording plate increased significantly in the stimulated hand after DBS electrode implantation [76]. Waldau et al. concluded that their VR glove may be useful as a quick intraoperative measurement to check correct electrode placement, complementing the clinician’s and patient’s subjective sense of improvement [78]. Wu et al. showed that intraoperative changes in bradykinesia can be evaluated with a noninvasive, objective, simple, and sensitive optical LMC device [90]. Yu et al. demonstrated how optical LMC measurements can be used during stereotactic neurosurgery to objectively assess bradykinesia, without interfering with voluntary movements [93]. The LMC was validated with a motion capture system, resulting in an average Pearson’s correlation coefficient of 0.986 [93].

#### Rigidity

Similar to bradykinesia, intraoperative assessment of rigidity has received less attention compared to tremor, as only five studies were included in the review. There was little overlap in devices and analysis techniques, ranging from accelerometry [84], gyroscopes [89], sEMG [74], to IMUs [80, 86].

Landy et al. showed that resting spontaneous EMG activity decreased if rigidity and/or tremor severity was reduced by lesioning. As such, sEMG may contribute to safe targeting in pallidotomy [74]. Kwon et al. showed that rigidity during DBS surgery can be quantified best by a biomechanical outcome measure called the viscous damping constant [80]. Shah et al. showed that 10 of 12 DBS-placement choices would have been different if accelerometric data was considered by the neurosurgeon, showing the potential of accelerometry to assess rigidity during stereotactic neurosurgery [84]. Schaeffer et al. underlined that long-term work is necessary to assess whether motor quantification alters intraoperative decision-making, and how this influences patient outcomes [86]. Múrias Lopes et al. concluded that their device supports the intraoperative evaluation of the effectiveness of DBS surgery and also foresees its application in pharmacological clinical trials [89].

## Discussion

This scoping review clearly shows that there are many intraoperative possibilities to implement objective measurement techniques to guide DBS or ablation. Based on the reported literature, there is no consensus on protocols nor analysis techniques. Nevertheless, most studies show that objective measurements can very well complement expert evaluation, are easily applicable, and have the potential to improve targeting in stereotactic neurosurgery.

Comparison of the results is challenging, since the study designs and technical considerations were widely diverse. The described studies have varying cohorts (Table 1) and make use of various outcome measures (Table 2–4). Also, a wide variety of analysis techniques was applied in the included studies, making it difficult to conclude which approach was superior. None of the studies directly compared the clinical outcome of sensor-based stereotactic neurosurgeries with the clinical outcome of standard procedure. Consequently, none were able to show that electronic assessment was more sensitive or accurate than expert assessment, or outperformed clinical assessment in any other way. Also, it was not shown that electrode placement based on objective assessment was superior in clinical effect or surgical outcome to placement based on expert assessment.

Unfortunately, most of the described electronic motor measurements lack standardized protocols. There is currently no international consensus on clinical protocols for intraoperative monitoring during stereotactic neurosurgery for movement disorders. Also, solid proof or validation regarding which outcome measures are superior in quantifying the intraoperative effect of stereotactic neurosurgery is still lacking [97]. Journee et al. described conditions and limitations for performing intraoperative tremor, bradykinesia, and rigidity measurements [49]. These aspects need to be considered when setting up a sensor-based approach for measuring motor symptoms in a clinical setting.

A possible reason that only one DT patient was included might be that stereotactic neurosurgery in dystonia patients is often performed under total anesthesia, which disallows intraoperative motor measurements. Also, the characteristics of DT are not as clearly defined as those in cases of parkinsonian and essential tremor, making it more difficult to quantify. Moreover, the interpretation of intraoperative assessments of DT is more challenging, since DT characteristics present higher variability compared to PD and ET [33, 34].

The inclusion of only two HT patients is probably caused by the low prevalence of this tremor syndrome [98–100]. Also, the indication for DBS is not clear yet for this patient population [101]. Moreover, HT is more difficult to measure electronically due to low-frequent proximal muscle contractions, as opposed to distal tremor syndromes like PD and ET [31].

A possible reason for the low number of included studies for bradykinesia and rigidity is that these are more complex symptoms to measure than tremor. Sensor techniques that have been validated for the quantification of these symptoms are often too intricate and invasive for application in a neurosurgical setting [22, 68].

It stood out that approaches in tremor-focused studies were more homogeneous than those for bradykinesia and rigidity, though studies on bradykinesia and rigidity disclosed less information on their techniques than tremor-focused studies. As these are more complex symptoms to analyze, their full data analysis protocol might have been too expansive for publication. Another explanation might be the use of patented devices and software, limiting public knowledge on how signal processing and data analysis was done. The use of closed-source systems, along with high costs and complexities in appliance, may contribute to the lack of standardized, open-source protocols for quantifying motor symptoms during stereotactic neurosurgery [86].

It stood out there was fewer information available about the usefulness and efficacy of intraoperative electronic measurements of bradykinesia than for tremor. Commercially available systems were used in all five studies that were included for the bradykinesia section. Although this may have limited the data analyses techniques presented by the authors, there are several studies outside the neurosurgical setting available to show the usefulness and efficacy of these methods to assess bradykinesia [21, 102].

Although the results of this review indicate that intraoperative clinical assessments might benefit from electronic motor measurements, some disadvantages are associated with these types of measurements (e.g., expenses, time investment, required technical knowledge). Additionally, objective measurements still need the interpretation of clinicians for clinical decision-making. Currently, it is not to be expected that objective methods for measuring motor symptoms will replace expert assessment. Nevertheless, these electronic methods can complement the current expert-approach, allowing objective input for clinical decision-making.

### Future perspectives

In this scoping review, the spectrum of electronic techniques that can be applied to an intraoperative setting to measure tremor, bradykinesia, and rigidity during awake stereotactic neurosurgery in Parkinson’s disease and tremor patients was described. In order to definitely prove the added value of their use, a multicenter or global collaboration aiming to validate electronic outcome measures and standardize objective intraoperative protocols is proposed. The open-source publication of these protocols and data analysis techniques is key here. Also, randomized controlled trials, involving multiple expert movement disorders neurologists, are suggested to investigate the added sensitivity and accuracy of electronic measurements to improve clinical assessment. All of this aiming to come to a consensus on the intraoperative application of these electronic measurements.

Although data-driven approaches described in this review offer interesting data and many different parameters, their use is often accompanied with complex hardware and software [21, 97]. For most techniques, sensor calibration and pre-processing of the data are intricate and time-consuming. So, technical expertise of these sensors and their data analysis is needed in order to come to data that is clinically useful. Since objective measurements result in complex quantitative data, their use calls for advanced data analysis to quickly evaluate large amounts of data, to identify classification patterns, and to present the data in a quickly interpretable manner. Machine learning algorithms and other mathematical procedures, such as principal component analysis, can be considered.

A number of techniques used intraoperatively can be used outside the operating room, for example, at the outpatient clinic or at the patient’s home. Electronic measurements can be applied preoperatively to aid prediction of surgery outcome and optimize selection of surgical candidates, as well as postoperatively to objectively monitor symptom severity at follow-up and to optimize DBS settings [103–108]. These are promising developments in the context of the uprise of closed-loop or adaptive DBS [109, 110]. Also, there is great potential in applying sensor-based monitoring at home to support clinicians in optimally managing patients between hospital appointments [111, 112]. Consistent use of accurate and objective methods to monitor motor symptoms throughout the caretaking process might contribute to the best possible clinical outcome. These quantitative measurements can complement clinical motor assessment in evaluating the efficacy of neurosurgical treatments [19].

## Conclusion

This scoping review provides a comprehensive overview of the most common electronic methods for intraoperatively measuring tremor, bradykinesia, and rigidity, showing that there are many possibilities to implement objective measurement techniques in the intraoperative setting. On the other hand, a lack of standardization was identified in this field. Although most included studies provided reliable outcome measures that correlated well with clinical assessments, sensor-based assessment was not shown to be more sensitive or accurate than expert assessment. Also, it was not proven that electrode placement based on objective assessment was superior to placement based on clinical assessment. Nevertheless, it is clear that objective measurements can complement visual evaluation of motor symptoms and may provide an objective confirmation of the surgical result. On the long term, this might benefit patient outcomes and provide reliable outcome measures in scientific research. For future research, it is suggested to develop standardized protocols with validated outcome measures that aid in predicting (long term) neurosurgical outcome.

## Data Availability

Not applicable.

## Appendix 1

### Search string from PubMed

("intraoperative*"[Title/Abstract] OR "intra-operative*"[Title/Abstract])

AND

("quanti*"[Title/Abstract] OR "assess*"[Title/Abstract] OR "measur*"[Title/Abstract])

AND

("Tremor"[Mesh] OR "Hypokinesia"[Mesh] OR "Muscle Rigidity"[Mesh] OR "tremor"[Title/Abstract] OR "bradykine*"[Title/Abstract] OR "rigid*"[Title/Abstract])

AND

("Accelerometry"[Mesh] OR "Acceleration"[Mesh] OR "Myography"[Mesh] OR "reliab*"[Title/Abstract] OR "valid*"[Title/Abstract] OR "mechanic*"[Title/Abstract] OR "accelero*"[Title/Abstract] OR "velo*"[Title/Abstract] OR "EMG"[Title/Abstract] OR "gyroscop*"[Title/Abstract] OR "kinematic*"[Title/Abstract] OR "motion sensor*"[Title/Abstract])

AND

("Deep Brain Stimulation"[Mesh] OR "DBS"[Title/Abstract] OR "neurosurg*"[Title/Abstract] OR "thalamotom*"[Title/Abstract] OR "pallidotom*"[Title/Abstract])

NOT

"animal"[Title/Abstract]
